# Current research and future directions of melatonin's role in seed germination

**DOI:** 10.1007/s44154-023-00139-5

**Published:** 2023-12-04

**Authors:** Ze Liu, Hengrui Dai, Jinjiang Hao, Rongrong Li, Xiaojun Pu, Miao Guan, Qi Chen

**Affiliations:** Faculty of Life Science and Technology, Kunming University of Science and Technology, Kunming, 650500 Yunnan China

**Keywords:** Melatonin, Seed germination, Normal conditions, Stressful conditions, ABA, GA, ROS, Metabolism

## Abstract

Seed germination is a complex process regulated by internal and external factors. Melatonin (*N*-acetyl-5-methoxytryptamine) is a ubiquitous signaling molecule, playing an important role in regulating seed germination under normal and stressful conditions. In this review, we aim to provide a comprehensive overview on melatonin's effects on seed germination on the basis of existing literature. Under normal conditions, exogenous high levels of melatonin can suppress or delay seed germination, suggesting that melatonin may play a role in maintaining seed dormancy and preventing premature germination. Conversely, under stressful conditions (e.g., high salinity, drought, and extreme temperatures), melatonin has been found to accelerate seed germination. Melatonin can modulate the expression of genes involved in ABA and GA metabolism, thereby influencing the balance of these hormones and affecting the ABA/GA ratio. Melatonin has been shown to modulate ROS accumulation and nutrient mobilization, which can impact the germination process. In conclusion, melatonin can inhibit germination under normal conditions while promoting germination under stressful conditions via regulating the ABA/GA ratios, ROS levels, and metabolic enzyme activity. Further research in this area will deepen our understanding of melatonin's intricate role in seed germination and may contribute to the development of improved seed treatments and agricultural practices.

## Introduction

Seed germination is a crucial stage in the life cycle of seed plants, which is indispensable for maintaining ecological balance, biodiversity, and maintaining ecosystem stability. Seed germination is defined as the initiation of the seed from water absorption and completion when the radicle protrudes from the seed coat (Ibrahim et al. 2021; Wolny et al. 2018). Both external conditions (e.g., moisture, temperature, abiotic stress, after-ripening) and internal factors (e.g., endogenous phytohormones, ROS) can affect seed germination (Carrera et al. 2008; Ibrahim et al. 2021).

Melatonin (*N*‐acetyl‐5‐methoxytrytamine) is a ubiquitous signaling molecule in plants and animals. The biosynthetic melatonin in plant cells is often called phytomelatonin, which regulates multiple plant growth stages and processes, including seed germination, stress response, and plant immunity. Phytomelatonin is synthesized using tryptophan as a precursor through four-step enzymatic reactions catalyzed by six biosynthesizing enzymes, namely tryptophan hydroxylase (TPH), tryptophan decarboxylase (TDC), tryptamine-5-hydroxylase (T5H), *N*-acetylserotonin methyltransferase (ASMT), caffeic acid-*O*-methyltransferase (COMT), serotonin-*N*-acetyltransferase (SNAT). Among them, SNAT and ASMT have been regarded as rate-limiting enzymes (Back 2021; Back et al. 2016). In 2018, the first phytomelatonin receptor (PMTR1) has been found in *Arabidopsis thaliana* (Wei et al. 2018). Further studies have found that PMTR1 is required for perceiving melatonin signaling in the regulation of stomatal closure and stomatal immunity, seed germination and seedling growth, flowering, resistance to drought, salt and high light stresses, and leaf senescence (Khan et al. 2023; Li et al. 2020a; Wei et al. 2018; Yang et al. 2021a; Yin et al. 2022).

In addition to its recognized importance as a plant hormone in plant development, melatonin also plays various roles in regulating seed germination under different conditions (Table 1). However, the role of melatonin in plant seed germination has not been well assessed. In this review, we appraise the literature on this nascent field, paying particular attention to the effects of melatonin on seed germination in both normal and stressful conditions. We further explore the interplay between abscisic acid (ABA), gibberellic acid (GA), reactive oxygen species (ROS), and melatonin in the regulation of seed germination. Finally, we provide an overview of melatonin's involvement in seed germination and propose future research directions to elucidate the molecular mechanisms of the melatonin signaling pathway and its potential implications in agricultural production.

**Table 1:** Effect of melatonin on seed germination under different conditions

Conditions	Species	Concentrations of melatonin (μM)	Effect on seed germination	Reference
**1/2MS**	*Arabidopsis thaliana*	500	inhibition	(Lv et al. 2021)
	*Arabidopsis thaliana*	10	no effcet	(Lv et al. 2021; Yin et al. 2022)
**Wet filter paper**	Stevia (*Stevia rebaudiana*)	500	inhibition	(Simlat et al. 2018)
	Stevia (*Stevia rebaudiana*)	5	promotion	(Simlat et al. 2018)
**Chilling stress**	Waxy Maize (*Zea mays*)	50	promotion	(Cao et al. 2019)
	Wheat(*Triticum aestivum*)	500 or 1000	promotion	(Li et al. 2018; Zhang et al. 2021a)
	Maize (*Zea mays*)	50	promotion	(Kolodziejczyk et al. 2021)
	Cucumber (*Cucumis sativus*)	10 or 25	promotion	(Posmyk et al. 2009; Zhang et al. 2022)
	Rice (*Oryza sativa*)	150	promotion	(Li et al. 2021b)
**Heat stress**	*Arabidopsis thaliana*	300	promotion	(Hernandez et al. 2015)
	Rice (*Oryza sativa*)	100	promotion	(Yu et al. 2022)
**Osmotic (or drought) stress**	Wheat (*Triticum aestivum*)	1 or 300	promotion	(Cui et al. 2018; Li, et al. 2020b)
	Cucumber (*Cucumis sativus*)	50	promotion	(Zhang et al. 2013)
	Cotton (*Gossypium hirsutum*)	100	promotion	(Bai et al. 2020)
	Triticale (*Triticale hexaploide*)	20	promotion	(Guo et al. 2022)
	Maize* (Zea mays)*	250	promotion	(Muhammad et al. 2023)
**Aged**	Maize* (Zea mays)*	10	promotion	(Deng et al. 2017)
	Oat (*Avena sativa*)	200	promotion	(Yan et al. 2020; Yan et al., 2021)
**Salt stress**	Wheat (*Triticum aestivum*)	1 or 300	promotion	(Wang et al. 2022; Zhang et al. 2021b)
	Halophyte sea lavender (*Limonium bicolor*)	1	promotion	(Li et al. 2019)
	Cotton (*Gossypium hirsutum*)	20	promotion	(Chen et al. 2020b, 2021)
	Alfalfa (*Medicago sativa*)	150	promotion	(Yu et al. 2021)
	Cucumber (*Cucumis sativus*)	1	promotion	(Zhang et al. 2014, 2017)
	Stevia (*Stevia rebaudiana*)	5	promotion	(Simlat et al. 2020)
	Tomato (*Solanum lycopersicum*)	*SlCOMT1* overexpression	promotion	(Ge et al. 2023)
	*Arabidopsis thaliana*	*VvSNAT1* overexpression	promotion	(Wu et al. 2021)
**Cr stress**	Wheat(*Triticum aestivum*)	100	promotion	(Lei et al. 2021)
	Rice (*Oryza sativa*)	50	promotion	(Li et al. 2022a)
**Cu stress**	Rice(*Oryza sativa*)	100	promotion	(Li et al. 2022b)
	Red cabbage (*Brassica oleracea rubrum*)	10	promotion	(Posmyk et al. 2008)
**Dark**	Almond (*Prunus dulcis*)	20	promotion	(García-Sánchez et al. 2022)
	Cotton (*Gossypium hirsutum*)	20	promotion	(Xiao et al. 2019)
**Soil**	Soybean(*Glycine max*)	50	promotion	(Wei et al. 2015)
**1 mM ABA**	Melon (*Cucumis melo*)	500	promotion	(Li et al. 2021b)

## Melatonin regulates seed germination under normal conditions

### High concentrations of melatonin inhibit seed germination

Melatonin can inhibit seed germination under normal conditions. Recently, Lv et al. (2021) found that exogenous melatonin application at low concentrations (e.g., 10 μM and 100 μM) had no effect on the germination of *Arabidopsis thaliana* seeds, while high concentrations (e.g., 500 μM and 1000 μM) of melatonin inhibited seed germination (Lv et al. 2021). Similarly, a high concentration (500 μM) of melatonin had a significant inhibitory effect on the germination of *Stevia rebaudiana* seeds compared with no melatonin treatment (Simlat et al. 2018). In addition, the modulation of melatonin biosynthesis genes using T-DNA insertion or over-expression alleles induces phenotypes consistent with melatonin playing a negative role in seed germination. For example, *Arabidopsis comt1* or *asmt* mutant seeds showed higher germination rates than the wild type (WT), while seeds from the *ASMT*-overexpressing plants showed a lower germination rate than WT. The inhibitory effect of melatonin on seed germination remained unaffected in the *pmtr1* mutant, indicating that PMTR1 may not be involved in the melatonin-mediated inhibition of seed germination (Lv et al. 2021). However, a more recent study by Yin et al. (2022) revealed contrasting findings. They found that the germination rate of the *pmtr1* mutant was higher compared to that of the WT. Conversely, the germination rate of *PMTR1*-overexpressing seeds was lower than that of the WT (Yin et al. 2022). Actually, the concentrations (500 and 1000 μM) of exogenous melatonin applied by Lv et al. (2021) were very high, exceeding the binding threshold of PMTR1. However, further investigation is necessary to determine the precise role of PMTR1 in the melatonin-mediated regulation of seed germination.

### Melatonin regulates seed germination by interacting with plant hormones ABA, GA and auxin

ABA and GA are two well-known hormones in regulating seed germination. ABA induces seed dormancy and inhibits seed germination, while GA breaks seed dormancy and promotes seed germination. In order to complete germination, the content of ABA decreases gradually while GA increases gradually after imbibition (Pan et al. 2021; Shu et al. 2016). Numerous studies have shown that seed germination depends on the balance between ABA and GA, rather than their absolute levels. An increase in the ABA/GA ratio inhibits seed germination, while a decrease in the ratio promotes it. (Chen et al. 2020a; Shu et al. 2016).

The biosynthesis and catabolism of ABA in plants are highly complex, involving multiple pathways that collectively determine ABA levels. In most plants, ABA biosynthesis begins with *β*-carotene and progresses through six or seven sequential steps to produce ABA. Several key enzymes catalyze the biosynthesis process, including Zeaxanthin Epoxidase (ZEP), 9-cis-epoxycarotenoid dioxygenase (NCEDs), ABA Deficiency 2 (Short Chain Alcohol Dehydrogenase, ABA2), and abscisic aldehyde oxidase 3 (AAO3). It is worth noting that NCEDs is regarded as a rate-limiting enzyme in this process (Schwartz et al. 2003; Yin et al. 2022). Alternatively, ABA catabolism involves the catalytic action of cytochrome P450s, specifically family 707 and subfamily A (CYP707As) (Ali et al. 2022; Yin et al. 2022). It has been proposed that the ABA content in seeds is primarily regulated by the expression of *NCEDs* and *CYP707As* genes (Tuan et al. 2018). GA is produced with geranylgeranyl diphosphate as a precursor though multiple reactions catalyzed by various enzymes. These enzymes include ent-copalyl diphosphate synthase (CPS), ent-kaurene synthase (KS), ent-kaurene oxidase (KO) and ent-kaurenoic acid oxidase (KAO) (Xie et al., 2020). The level of bioactive GA depends on the balance between their biosynthesis and deactivation. The biosynthesis of GA is mainly catalyzed by GA20 oxidase (GA20ox) and GA3 oxidase (GA3ox), and their deactivation is mainly controlled by GA2 oxidase (GA2ox) (Tuan et al. 2018).

In *Arabidopsis thaliana*, Lv et al. (2021) found that high concentrations of exogenous melatonin upregulated ABA biosynthesis genes in seeds, including *NCED3* and *ABA2*, resulting in increased ABA contents. However, the impact on GA was minimal, resulting in an elevated ABA/GA ratio, which ultimately inhibited seed germination (Lv et al. 2021). Furthermore, melatonin exhibited a significant enhancement in the expression of *ABI3* and *ABI5*, indicating that melatonin partially governs seed germination through the ABA pathways mediated by ABI3 and ABI5 (Lv et al. 2021). Consistent with this finding, the expression of *ABI5* in the seeds of the *asmt* mutant was observed to be lower compared to the WT. Conversely, the *ABI5* expression in *ASMT*-overexpressing seeds was higher than that in the WT (Lv et al. 2021). Moreover, Yin et al. (2022) demonstrated that expression levels of ABA catabolism genes (*CYP707A1-A4*) in dry seeds of the *pmtr1* mutant were significantly higher than that of the WT. Conversely, the expression of the ABA catabolic gene in overexpressing-*PMTR1* seeds was significantly lower than in the WT. Consequently, the ABA contents in the *pmtr1* mutant seeds were lower than in the WT, whereas the ABA contents in *PMTR1*-overexpressing seeds were higher than in the WT (Yin et al. 2022). These findings suggest that PMTR1 may play a role in regulating ABA catabolism during seed development and germination in *Arabidopsis thaliana*.

Auxin is another indole derivative that shares the same synthetic precursor, tryptophan, with the melatonin (Arnao et al., 2014; Lv et al. 2021). Auxin has previously been shown to inhibit *Arabidopsis thaliana* seed germination in an ABA-dependent manner (Liu et al. 2013). Interestingly, Lv et al. (2021) reported that exogenous IAA can alleviate the inhibition of melatonin on seed germination. Furthermore, melatonin was found to increase contents of IAA in *Arabidopsis thaliana* seeds via upregulating expression of the IAA response genes (e.g., *IAA3* and *IAA13*) (Lv et al. 2021). This could be attributed to the competitive mechanism between endogenous IAA and melatonin biosynthesis following treatment with melatonin or IAA. These findings suggest that melatonin plays a similar role to auxin in inhibiting seed germination. However, an antagonistic competition may also occur in their intracellular signaling pathways.

ABA, GA, and auxin exhibit complex crosstalk with melatonin during seed germination. Melatonin and ABA act synergistically to inhibit seed germination, while melatonin and GA have opposing effects: GA promotes seed germination, while melatonin inhibits it. Additionally, melatonin and auxin counteract each other, with auxin enhancing the inhibitory effect of melatonin on seed germination (Lv et al. 2021). In the regulation of seed germination, several other plant hormones also play significant roles. For example, auxin and jasmonic acid could inhibit seed germination, while ethylene and brassinosteroids have promotion effects (Ibrahim et al. 2021; Pan et al. 2023; Steber et al., 2001; Wang et al. 2020). Crosstalk between melatonin and other phytohormones in non-seed germination stage has been reported. For example, melatonin has been shown to regulate auxin biosynthesis and signaling for plant root development (Liang et al. 2017; Yang et al. 2021b). Melatonin enhances postharvest disease resistance in blueberry fruit by modulating the jasmonic acid signaling pathway (Qu et al. 2022). Melatonin treatment reduces ethylene production and helps maintain apple fruit quality during post-harvest storage (Onik et al. 2021). Additionally, melatonin plays an active role in regulating growth in dark or shaded conditions by affecting brassinosteroid biosynthesis (Hwang and Back 2018). However, the question of whether crosstalk between melatonin and other plant hormones occurs during seed germination remains to be explored.

### Melatonin affects seed germination by regulating ROS

Reactive Oxygen Species (ROS) are proposed as "Oxidative Window for Germination" to initiate seed germination and release seed dormancy (Bailly et al. 2008; Waszczak et al. 2018). They are mainly produced by the mitochondrial electron transport chain and NADPH oxidase (also known as respiratory burst oxidase homologues, RBOHs) after seed imbibition and prior to radical protrusion (Bailly 2019; Jurdak et al. 2021). The external application of H_2_O_2_ can break seed dormancy and promote seed germination (Liu et al. 2010). To complete germination successfully, seeds need to accumulate high levels of ROS in a very short time. Thereafter ROS are dramatically decreased to function as signaling molecules in regulating plant growth (Bailly 2019; Leymarie et al. 2012; Liu et al. 2010). On the other hand, ROS are commonly considered as toxic compounds because they can react with almost all biological molecules, including lipids, nucleic acids and proteins, causing severe cellular and biological damage (Demidchik 2015; Mittler 2002). Due to this dual role, Jurdak et al. (2022) proposed that seed germination can only occur when ROS contents are controlled within a suitable range to ensure ROS signaling rather than ROS damage. Either too low or too high ROS levels would impair seed germination (Jurdak et al. 2022). During seed germination, appropriate ROS can cross talk with other phytohormones, mainly ABA and GA. Some studies have shown that the accumulation of H_2_O_2_ in seeds can increase germination and decrease ABA contents by up-regulating the genes (*CYP707As*) responsible for ABA catabolism (Liu et al. 2010). It has also been demonstrated that ROS stimulates GA biosynthesis by up-regulating genes such as *GA3OX1* and *GA20OX1* (Kai et al. 2016; Liu et al. 2010).

**Fig. 1:**
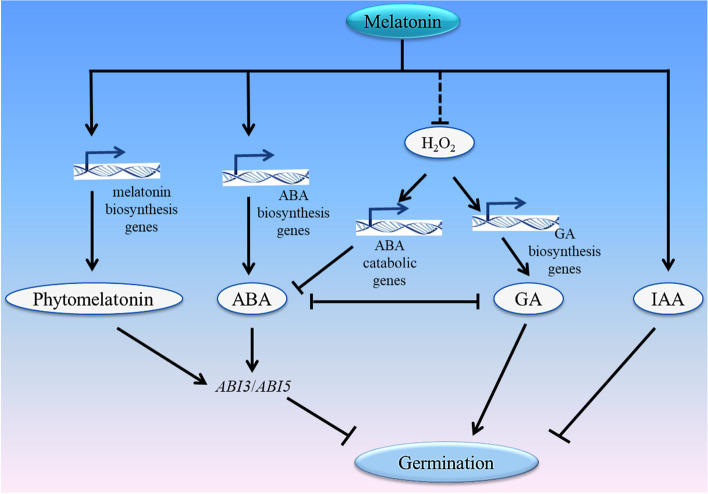
Melatonin inhibits seed germination under normal conditions. Exogenous melatonin can promote the biosynthesis of endogenous melatonin, and endogenous melatonin can enhance the expression of *ABI5*. Melatonin promotes ABA biosynthesis by up-regulating the expression of ABA biosynthesis genes such as *NCEDs*, thereby enhances *ABI5* and *ABI3* expression. As an antioxidant, melatonin may reduce the amount of H_2_O_2_. H_2_O_2_ accelerates the breakdown of ABA by up-regulating catabolic genes, such as *CYP707As*. H_2_O_2_ promotes GA biosynthesis by up-regulating the expression of GA biosynthesis genes, such as *KS*. Melatonin can promote IAA biosynthesis and signaling. ABA and IAA inhibited seed germination. GA and H_2_O_2_ promoted seed germination. The arrows represent promotion, the short lines represent inhibition, and the dashed lines represent possible pathways

Melatonin, a widely recognized antioxidant, has been demonstrated to activate the plant's antioxidant system to eliminate ROS (Bajwa et al. 2014; Lu et al. 2022). Therefore, when melatonin is applied under normal germination conditions, ROS accumulation in seeds can decrease to excessively low levels, which may hinder efficient germination. Simultaneously, inadequate ROS accumulation can hinder the stimulatory effects of ROS on ABA catabolism and GA biosynthesis, leading to an elevated ABA/GA ratio that inhibits seed germination (Fig. 1). However, during seed germination, it remains unclear whether high concentrations of melatonin directly increase ABA biosynthesis or whether melatonin first reduces the level of ROS, thereby inhibiting subsequent the ROS-mediated regulation of ABA catabolism.

## Melatonin promotes seed germination under stressful conditions

### Melatonin promotes seed germination by regulating ABA and GA

Stressful germination conditions can lead to an increase or a decrease in ABA or GA levels in seeds, respectively. For instance, under salt stress, soybean seeds accumulated high levels of ABA due to the up-regulation of ABA biosynthesis genes *GmNCEDs* and *GmAAO*, while GA levels were reduced by the down-regulation of GA biosynthesis genes *GmKAO* and *GmGA3OX1*, as well as the up-regulation of a GA catabolic gene, *GmGA2OX8* (Shu et al. 2017). However, melatonin can decrease ABA levels and increase GA levels, thus reversing the consequences caused by stress treatment and ensuring seed germination. When 10 μM of melatonin was applied to cotton seeds germinating under salt stress, lower levels of ABA and higher levels of GA were observed in the seeds compared to those without melatonin treatments. Similar results were consistently found in rice seeds germinating under chromium and low-temperature stress, in cucumber seeds germinating under low-temperature stress, in *Limonium bicolor* seeds germinating under salt stress (Li et al. 2019, 2021a, 2022a; Zhang et al. 2014, 2022). In addition, the reduction of ABA contents through melatonin application under stressful conditions was demonstrated to be involved in the down-regulation of ABA biosynthesis genes *NCEDs* (e.g., *NCED1*, *NCED3*) and the up-regulation of ABA catabolic genes *CYP707As* (e.g., *CYP707A1*, *CYP707A2*) (Li et al. 2019; Zhang et al. 2014, 2022). Regarding GA, researchers have discovered that the increase in GA contents in seeds under salt stress was attributed to the up-regulation of the GA biosynthesis genes *GA20OX* and *GA3OX* (Li et al. 2019; Zhang et al. 2014, 2022). Consistent with the information provided, the overexpression of melatonin biosynthesis genes can also lead to the phenotype of reduced ABA levels and increased GA levels for seed germination under stressful conditions. For instance, Ge et al. (2023) engineered a *SlCOMT*-overexpressing *Solanum lycopersicum L*. to enhance melatonin contents in the transgenic seeds. In comparison to WT, the transgenic seeds exhibited decreased ABA contents, which were attributed to down-regulation of an ABA biosynthesis gene *SlABA1* and the up-regulation of an ABA catabolic gene, *SlCYP707A1*. Meanwhile, the GA contents were elevated in the transgenic plants due to the up-regulation of a GA biosynthesis gene, *SlGA3OX*. Consequently, these findings contribute to enhanced salt tolerance and improved seed germination in the *SlCOMT*-overexpressing plant (Ge et al. 2023). Similarly, seeds of the *VvSNAT1*-overexpressing *Arabidopsis thaliana* exhibited enhanced tolerance to salt stress and faster germination in comparing to the WT (Wu et al. 2021).

Melatonin also plays an indirect role in the regulation of ABA signaling components, facilitating effective control of seed germination under stressful conditions. For example, treatment with melatonin has been shown to reduce the ABA content in cucumber seeds exposed to low temperatures. In this context, the ABA receptor PYR/PYL/RCAR is unable to bind to ABA, thus inhibiting the binding of CsPYL to CsPP2C. Consequently, this leads to an increase in CsPP2C activity, while simultaneously blocking the activation of CsSnRK2.1 and the phosphorylation of downstream factors such as ABA responsive element binding factor (ABF)/ABA responsive element binding protein (AREB) by CsSnRK2.1 (Zhang et al. 2022). In a separate study, Li et al. (2022b) found that the rice mutant *abi5*, which lacks the ability to respond to melatonin-induced relief from Cr stress, suggests a potential relationship between ABI5 and the alleviation of Cr stress by melatonin (Li et al. 2022b).

### Melatonin promotes seed germination by removing excessive ROS

A number of studies have shown that plants can produce a significant amount of ROS (including H_2_O_2_, O^2^^-^, OH^−^, etc.) when growing under stressful conditions (Lin et al. 2013; Liu et al. 2019; Luo et al. 2021). Unlike their signaling function under normal conditions that promote seed germination, ROS produced under stress can cause damage to plant cells and are not conducive to plant growth and development.

Melatonin has been regarded as a master regulator of ROS signaling (Chen et al., 2022; Wei et al. 2018). When plants are grown under stressful conditions like salt stress, drought stress, UV-B exposure, and others, melatonin can rapidly activate the antioxidant systems. This activation helps to eliminate excess ROS and reduce oxidative damage within the plants (Fan et al. 2018; Hassan et al. 2022; Liu et al. 2022; Sharif et al. 2018; Yao et al. 2021). For instance, melatonin treatment was observed to enhance the germination of waxy maize under cold stress (Cao et al. 2019). Concurrently, the activities of several antioxidant enzymes, including superoxide dismutase (SOD), peroxidase (POD), catalase (CAT), and ascorbate peroxidase (APX), were significantly increased in the group treated with melatonin. Furthermore, the levels of H_2_O_2_ and malondialdehyde (MDA) produced as results of cold stress were significantly decreased. This demonstrates the role of melatonin in mitigating oxidative stress and supporting seed germination in stressful conditions (Cao et al. 2019). Kolodziejczyk et al. (2021) revealed that melatonin has the capability to enhance maize seed germination under cold stress (Kolodziejczyk et al. 2021). This improvement is attributed to melatonin's ability to elevate the activities of glutathione (GST) and glutathione reductase (GSSG-R). These findings suggest that melatonin plays a role in boosting the antioxidative defense mechanisms in maize seeds subjected to cold stress, thereby aiding in the germination process (Kolodziejczyk et al. 2021).

### Melatonin promotes seed germination by regulating nutrient mobilization in seeds

Desiccated seeds are rich in storage substances, such as starch, storage proteins, and lipids. During seed germination, these substances are broken down and efficiently reutilized to provide the energy required for the initiating of germination and to facilitate a smooth transition into early seedling growth. In seeds, the primary enzymes responsible for starch breakdown are *α*-amylase and *β*-amylase. Among these, *α*-amylase plays a crucial role in remobilizing starch from the endosperm. It achieves this by hydrolyzing *α*-1, 4-glucan bonds within starch, converting them into amylose (Damaris et al. 2019; Zeeman et al. 2007). This metabolic process generates transportable nutrients, such as maltose and glucose, which play a crucial role in supporting seedling growth (Damaris et al. 2019; Krasensky et al., 2012) These nutrients are closely associated with both seed germination and the ability of plants to tolerate a range of abiotic stresses, such as drought, salinity, and extreme temperatures. On the other hand, during seed germination, the storage proteins need to be hydrolyzed by protease into free amino acids, for recompositing into new proteins to perform their function (Yu et al. 2015). Triacylglycerol (TAG), as the main storage lipid in seeds, needs to be catalyzed by TAG lipase to release fatty acids to produce acetyl-CoA that performs important biological roles in plants (Goepfert et al., 2007; Huang et al. 2021; Shrestha et al. 2016).

With respect to the utilization of seed storage for germination, ABA has been found to counteract the effects of GA. For instance, ABA can inhibit the metabolism of seed reserves (Tonini et al. 2010). However, GA can activate the expression of the *α*-amylase gene through the GA response element (GARC) in the gene promoter. This activation leads to the biosynthesis of *α*-amylase in seeds, facilitating the breakdown of starch (Lanahan et al. 1992; Shaik et al. 2014).

Melatonin has been proven to enhance the utilization of seed storage during germination under stressful conditions. For instance, the application of melatonin under Cr stress significantly increased the content of soluble sugars produced from starch breakdown in wheat seeds (Lei et al. 2021). This increase was accompanied by an improved activity of *α*-amylase. Furthermore, the seeds treated with melatonin exhibited higher contents of free amino acids resulting from proteolysis, compared to the group without melatonin application (Lei et al. 2021). Similarly, Cao et al. (2019) found that melatonin can enhance the activity of *α*-amylase and *β-*amylase in waxy maize seeds under cold stress, thereby promoting starch hydrolysis, leading to an increase in soluble and reducing sugar contents, and ultimately promoting seed germination (Cao et al. 2019). Zhang et al. (2017) employed a forward proteomics approach to investigate the mechanisms through which melatonin promotes cucumber seed germination under salt stress (Zhang et al. 2017). Their findings revealed that melatonin not only enhances the expression of anti-stress proteins within the seeds, but also substantially increases the expression of proteins involved in ATP production for energy metabolism across glycolysis, the citric acid cycle, and the glyoxylic acid cycle. Additionally, their research indicated that melatonin treatment significantly reduces the content of fatty acids, which are pivotal in mediating the utilization of seed reserves. This reduction suggests that melatonin facilitates the catabolism of fatty acids, thus providing the necessary energy for stress resistance during seed germination (Zhang et al. 2017). The phenomenon of increased seed reserve utilization during germination under stressful conditions can also be induced by overexpressing the melatonin biosynthesis gene *COMT*. Ge et al. (2023) developed *Solanum lycopersicum L*. plants with overexpressed *SlCOMT*, which resulted in seeds displaying enhanced germination under salt stress. This enhancement was attributed to the increased activity of amylase, alongside elevated levels of soluble sugars and proline within the seeds (Ge et al. 2023). These findings clearly indicate that melatonin can improve the utilization of stored substances in seeds by upregulating the activity of related catabolic enzymes, thereby improving seed germination under stressful conditions.

The exact mechanism by which melatonin regulates the activity of substance metabolism enzymes is not yet fully understood. It was speculated that melatonin treatment could increase the content of GA, leading to the upregulation of α-amylase genes (Lei et al. 2021; Damaris et al. 2019). On the other hand, Zhao et al. (2015) demonstrated that melatonin directly upregulates the expression of the *SUS2* gene in maize. The enzyme encoded by the *SUS2* gene is responsible for the decomposition of sucrose (Zhao et al. 2015). Nevertheless, further research is needed to shed light on the precise mechanisms underlying melatonin's regulation of substance metabolism enzymes.

## Conclusions and prospectives

**Fig. 2:**
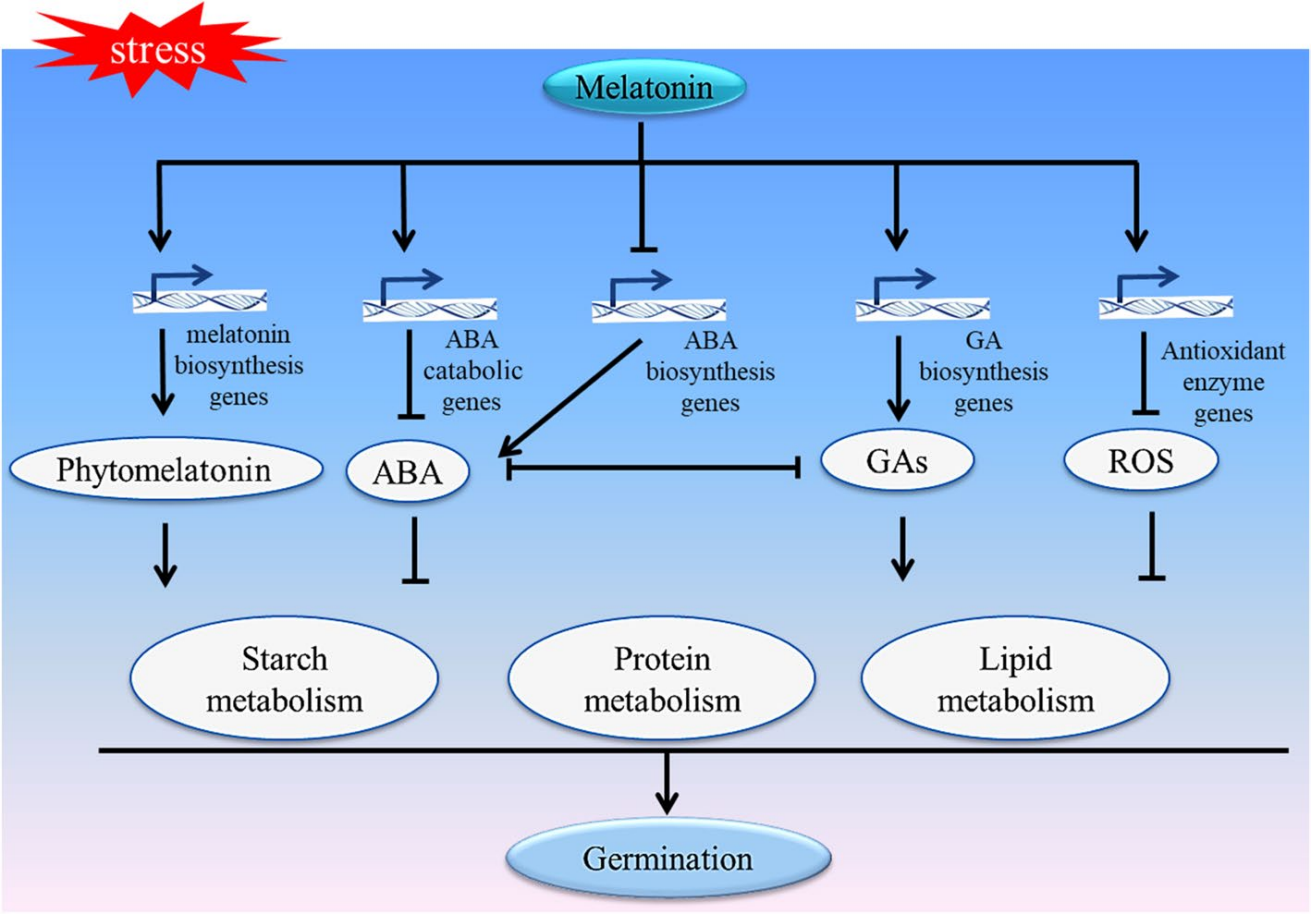
Melatonin promotes seed germination under stressful conditions. Briefly, melatonin can improve the internal conditions of seeds by regulating the level of plant hormones ABA and GA, and removing extra ROS from seeds to reduce the oxidative damage. In addition, exogenous melatonin can promote the biosynthesis of endogenous melatonin, and endogenous melatonin promotes the mobilization of nutrients in seeds in a couple of ways: 1) melatonin down-regulates the expressions of ABA biosynthesis genes (such as *NCEDs*) and up-regulates the expressions of ABA catabolic genes (*CYP707As*), thus reduces the ABA content to alleviate the inhibitory effect of ABA on the mobilization of nutrients in seeds; 2) melatonin promotes the GAs biosynthesis by up-regulating the expressions of GAs biosynthesis genes (such as *GA3ox*), and the positive effect of GAs on nutrient mobilization in seeds can be enhanced; 3) As an antioxidant, melatonin reduces ROS by improving the activity of antioxidant enzymes (such as SOD and POD), to decrease the oxidative damage aroused from stressful conditions to seeds, and ensures efficient mobilization of nutrients in seeds. The arrows represent promotion, the short lines represent inhibition

Seed germination is a highly complex process that is influenced by multiple factors. Melatonin, a versatile signaling molecule, plays a regulatory role in the balance of ABA/GA and ROS contents in seeds during seed germination. This in turn affects substance metabolism and regulates seed germination. Under normal germination conditions, melatonin upregulates the expression of ABA biosynthesis genes, leading to an increase in the ABA/GA ratio. Additionally, exogenous high levels of melatonin have the potential to decrease the levels of reactive oxygen species (ROS) below the normal range. This reduction in ROS contents obstructs the downward transmission of ROS signals, ultimately resulting in the inhibition of seed germination (Fig. 1). Under stressful germination conditions, melatonin reduces the ratio of ABA/GA by downregulating the expression of ABA biosynthesis genes and upregulating the expression of GA biosynthesis genes and ABA catabolism genes. Simultaneously, melatonin reduces the ROS contents induced by stress through the upregulation of antioxidant enzyme genes. This promotes material metabolism in seeds, ultimately facilitating seed germination (Fig. 2).

In recent years, the regulatory role of melatonin in seed germination has been continuously studied. However, the specific regulatory mechanisms are largely unknown. (1) There is evidence suggesting that the phytomelatonin receptor PMTR1 plays a negative regulatory role in seed germination in *Arabidopsis thaliana* (Yin et al., 2022a). However, Yu et al. (2021) reported that MsPMTR1 is required for the melatonin-promoted alfalfa seed germination under salt stress conditions (Yu et al. 2021). Nevertheless, it remains unclear whether melatonin relies on its receptor PMTR1 to directly participate in the regulation of seed germination under both normal and stressful conditions, and the underlying mechanism behind this interaction remains unknown. (2) In addition to ABA and GA, several other plant hormones are involved in the regulation of seed germination. Crosstalk between melatonin and other phytohormones has been reported in non-seed germination stages. However, it remains unclear whether there is crosstalk between melatonin and other plant hormones specifically during seed germination. If such crosstalk exists, the underlying mechanisms are yet to be elucidated. (3) Crop growth faces challenges due to climate change; whereas, melatonin shows potential in enhancing both the growth and yield of crops under stressful conditions. Several studies have shown that seed soaking with melatonin could promote seed germination, growth, yield and stress resistance of soybean, wheat and maize (Ahmad et al. 2021; Lei et al. 2021; Wei et al. 2015; Ye et al. 2020). Therefore, the breakthroughs in these questions will improve our understanding of the molecular mechanism by which melatonin regulates seed germination and provide insights into the application of melatonin in agricultural production.

## Data Availability

Not applicable.

## Abbreviations

ABA: Abscisic acid
GA: Gibberellin
ROS: Reactive oxygen species
NADPH: Reduced coenzyme II
